# Supplementary material for: A plasmid-based Escherichia coli gene expression system with cell-to-cell variation below the extrinsic noise limit
Source: PLoS One. 2017 Oct 30;12(10):e0187259. doi: 10.1371/journal.pone.0187259 (PMC5662224; doi:10.1371/journal.pone.0187259)
Supplement: S1 Text — DNA sequences for all constructs, Tables A and B, Figures A—F. (PDF) [file pone.0187259.s001.pdf]

A plasmid-based *Escherichia coli* gene expression system with cell-to-cell variation below the extrinsic noise limit

Zach Hensel[1,2]*

[1] Instituto de Tecnologia Química e Biológica António Xavier, Universidade Nova de Lisboa, Av. da República, 2780-157 Oeiras, Portugal
[2] Dean's Research Unit, Okinawa Institute of Science and Technology, Okinawa 904-0495, Japan

* For correspondence: zach.hensel@itqb.unl.pt

Supporting Material

DNA sequence for bicistronic, autoregulatory sequence. The synthetic sequence incorporated into pZH509 is shown below. Transcription start and translation start/stop signals are capitalized and bolded and other features are marked as indicated:

tccctatcagtgatagagattgacatccctatcagtgatagagatactgagcac**A**tcagcaggacgcactgaccg
aattcattaaagaggagaaaggtaccgc**ATG**agtaaaggagaagaacttttcactggagttgtcccaattcttgt
tgaattagatggtgatgttaatgggcacaaattttctgtcagtggagagggtgaaggtgatgcaacatacggaaa
acttacccttaaatttatttgcactactggaaaactacctgttccatggccaacacttgtcactactttcgcgta
tggtcttcaatgctttgcgagatacccagatcatatgaaacagcatgactttttcaagagtgccatgcccgaagg
ttatgtacaggaaagaactatatttttcaaagatgacgggaactacaagacacgtgctgaagtcaagtttgaagg
tgatacccttgttaatagaatcgagttaaaaggtattgattttaaagaagatggaaacattcttggacacaaatt
ggaatacaactataactcacacaatgtatacatcatggcagacaaacaaaagaatggaatcaaagttaacttcaa
aattagacacaacattgaagatggaagcgttcaactagcagaccattatcaacaaaatactccaattggcgatgg
ccctgtccttttaccagacaaccattacctgtccacacaatctgccctttcgaaagatcccaacgaaaagagaga
ccacatggtccttcttgagtttgtaacagctgctgggattacacatggcatggatgaactatacaaa**TAATAA**tc
tagcaggaggatttcacc**ATG**tctagattagataaaagtaaagtgattaacagcgcattagagctgcttaatgag
gtcggaatcgaaggtttaacaacccgtaaactcgcccagaagctaggtgtagagcagcctacattgtattggcat
gtaaaaaataagcgggctttgctcgacgccttagccattgagatgttagataggcaccatactcacttttgccct
ttagaaggggaaagctggcaagattttttacgtaataacgctaaaagttttagatgtgctttactaagtcatcgc
gatggagcaaaagtacatttaggtacacggcctacagaaaaacagtatgaaactctcgaaaatcaattagccttt
ttatgccaacaaggtttttcactagagaatgcattatatgcactcagcgctgtggggcattttactttaggttgc
gtattggaagatcaagagcatcaagtcgctaaagaagaaagggaaacacctactactgatagtatgccgccatta
ttacgacaagctatcgaattatttgatcaccaaggtgcagagccagccttcttattcggccttgaattgatcatc
tgcggattagaaaaacaacttaaatgtgaaagtgggtct**TAATAA**ctgcagcccggggggatcccatggtacgcgt
gctagaggcatcaaataaaacgaaaggctcagtcgaaagactgggcctttcgttttat

TetO2 GFPmut2 TetR *rrnB* T1

DNA sequences for "constitutive expression" (pZH514, pZH515, pZH516). The sequence is shown for pZH514 and annotated as above. The same mutated ribosome binding sites used for pZH511 (weak RBS) and pZH512 (moderate RBS) are used in pZH515 and pZH516, respectively.

tccctatcagtgatagagattgacatccctatcagtgatagagatactgagcac**A**tcagcaggacgcactgaccg
aattcattaaagaggagaaaggtaccgc**ATG**agtaaaggagaagaacttttcactggagttgtcccaattcttgt
tgaattagatggtgatgttaatgggcacaaattttctgtcagtggagagggtgaaggtgatgcaacatacggaaa
acttacccttaaatttatttgcactactggaaaactacctgttccatggccaacacttgtcactactttcgcgta
tggtcttcaatgctttgcgagatacccagatcatatgaaacagcatgactttttcaagagtgccatgcccgaagg
ttatgtacaggaaagaactatatttttcaaagatgacgggaactacaagacacgtgctgaagtcaagtttgaagg
tgatacccttgttaatagaatcgagttaaaaggtattgattttaaagaagatggaaacattcttggacacaaatt
ggaatacaactataactcacacaatgtatacatcatggcagacaaacaaaagaatggaatcaaagttaacttcaa
aattagacacaacattgaagatggaagcgttcaactagcagaccattatcaacaaaatactccaattggcgatgg
ccctgtccttttaccagacaaccattacctgtccacacaatctgccctttcgaaagatcccaacgaaaagagaga
ccacatggtccttcttgagtttgtaacagctgctgggattacacatggcatggatgaactatacaaa**TAATAA**tc
tagccatggtacgcgtgctagaggcatcaaataaaacgaaaggctcagtcgaaagactgggcctttcgttttat

TetO2 GFPmut2 *rrnB* T1

DNA sequences for "autoregulated repressor" (pZH517, pZH518, pZH519). The sequence is shown for pZH517 and annotated as above. The same mutated ribosome binding sites used for pZH511 (weak RBS) and pZH512 (moderate RBS) are used in pZH518 and pZH519, respectively.

tccctatcagtgatagagattgacatccctatcagtgatagagatactgagcac**A**tcagcaggacgcactgaccg
aattcattaaagaggagaaaggtaccgc**ATG**agtaaaggagaagaacttttcactggagttgtcccaattcttgt
tgaattagatggtgatgttaatgggcacaaattttctgtcagtggagagggtgaaggtgatgcaacatacggaaa
acttacccttaaatttatttgcactactggaaaactacctgttccatggccaacacttgtcactactttcgcgta
tggtcttcaatgctttgcgagatacccagatcatatgaaacagcatgactttttcaagagtgccatgcccgaagg
ttatgtacaggaaagaactatatttttcaaagatgacgggaactacaagacacgtgctgaagtcaagtttgaagg
tgataccctgttaatagaatcgagttaaaaggtattgattttaaagaagatggaaacattcttggacacaaatt
ggaatacaactataactcacacaatgtatacatcatggcagacaaacaaaagaatggaatcaaagttaacttcaa
aattagacacaacattgaagatggaagcgttcaactagcagaccattatcaacaaaatactccaattggcgatgg
ccctgtccttttaccagacaaccattacctgtccacacaatctgccctttcgaaagatcccaacgaaaagagaga
ccacatggtccttcttgagtttgtaacagctgctgggattacacatggcatggatgaactatacaaa**TAATAA**tc
tagcataaaacgaaaggctcagtcgaaagactgggcctttcgttttattccctatcagtgatagagattgacatc
cctatcagtgatagagatactgagcac**A**tcagcaggacgcactgaccaggaggatttcacc**ATG**tctagattaga
taaaagtaaagtgattaacagcgcattagagctgcttaatgaggtcggaatcgaaggtttaacaacccgtaaact
cgcccagaagctaggtgtagagcagcctacattgtattggcatgtaaaaaataagcgggctttgctcgacgcctt
agccattgagatgttagataggcaccatactcacttttgccctttagaaggggaaagctggcaagattttttacg
taataacgctaaaagttttagatgtgctttactaagtcatcgcgatggagcaaaagtacatttaggtacacggcc
tacagaaaaacagtatgaaactctcgaaaatcaattagccttttatgccaacaaggtttttcactagagaatgc
attatatgcactcagcgctgtggggcattttactttaggttgcgtattggaagatcaagagcatcaagtcgctaa
agaagaaagggaaacacctactactgatagtatgccgccattattacgacaagctatcgaattatttgatcacca
aggtgcagagccagccttcttattcggccttgaattgatcatctgcggattagaaaaacaacttaaatgtgaaag
tgggtct**TAATAA**ctgcagcccggggatcccatggtacgcgtgctagaggcatcaaataaaacgaaaggctcag
tcgaaagactgggcctttcgttttat

TetO2   GFPmut2   TetR   *rrnB* T1

DNA sequences for “constitutive repressor” (pZH520, pZH521, pZH522). The sequence is shown for pZH520 and annotated as above. The same mutated ribosome binding sites used for pZH511 (weak RBS) and pZH512 (moderate RBS) are used in pZH521 and pZH522, respectively.

tccctatcagtgatagagattgacatccctatcagtgatagagatactgagcac**A**tcagcaggacgcactgaccg
aattcattaaagaggagaaaggtaccgc**ATG**agtaaaggagaagaacttttcactggagttgtcccaattcttgt
tgaattagatggtgatgttaatgggcacaaattttctgtcagtggagagggtgaaggtgatgcaacatacggaaa
acttacccttaaatttatttgcactactggaaaactacctgttccatggccaacacttgtcactactttcgcgta
tggtcttcaatgctttgcgagatacccagatcatatgaaacagcatgactttttcaagagtgccatgcccgaagg
ttatgtacaggaaagaactatatttttcaaagatgacgggaactacaagacacgtgctgaagtcaagtttgaagg
tgatacccttgttaatagaatcgagttaaaaggtattgattttaaagaagatggaaacattcttggacacaaatt
ggaatacaactataactcacacaatgtatacatcatggcagacaaacaaaagaatggaatcaaagttaacttcaa
aattagacacaacattgaagatggaagcgttcaactagcagaccattatcaacaaaatactccaattggcgatgg
ccctgtccttttaccagacaaccattacctgtccacacaatctgccctttcgaaagatcccaacgaaaagagaga
ccacatggtccttcttgagtttgtaacagctgctgggattacacatggcatggatgaactatacaaa**TAATAA**tc
tagcataaaacgaaaggctcagtcgaaagactgggcctttcgttttatcacagctaacaccacgtcgtccctatc
tgctgccctaggtctatgagtggttgctggataactttacgggcatgcataaggctcgtaatatatattcaggga
gaccacaacggtttccctctacaaataatttgtttaactttaggaggatttcacc**ATG**tctagattagataaaa
gtaaagtgattaacagcgcattagagctgcttaatgaggtcggaatcgaaggtttaacaacccgtaaactcgccc
agaagctaggtgtagagcagcctacattgtattggcatgtaaaaaataagcgggctttgctcgacgccttagcca
ttgagatgttagataggcaccatactcacttttgccctttagaaggggaaagctggcaagattttttacgtaata
acgctaaaagttttagatgtgctttactaagtcatcgcgatggagcaaaagtacatttaggtacacggcctacag
aaaaacagtatgaaactctcgaaaatcaattagccttttatgccaacaaggtttttcactagagaatgcattat
atgcactcagcgctgtgggcattttactttaggttgcgtattggaagatcaagagcatcaagtcgctaaagaag
aaagggaaacacctactactgatagtatgccgccattattacgacaagctatcgaattatttgatcaccaaggtg
cagagccagccttcttattcggccttgaattgatcatctgcggattagaaaaacaacttaaatgtgaaagtgggt
ct**TAATAA**ctgcagcccgggggatcccatggtacgcgtgctagaggcatcaaataaaacgaaaggctcagtcgaa
agactgggcctttcgttttat

TetO2 GFPmut2 *rrnB* T1 proB TetR

Supplemental Tables

Table A. Ribosome binding site strength estimates compared to GFP expression levels (induction with 8 nM ATc) measured by flow cytometry (**Fig 3B**). GFP start codon and mutations from pZH509 sequence are capitalized.

Plasmid	RBS sequence	RBS strength	GFP expression	Ratio
pZH509	`aaagaggagaaaggtaccgcATG`	12,905	1,175.5	10.98
pZH510	`aaaTaggagaaaggtaccgcATG`	7,189	998.2	7.20
pZH513	`aaaCTCgagaaaggtaccgcATG`	3,307	644.3	5.13
pZH512	`aaagCCgagaaaggtaccgcATG`	2,016	509.6	3.96
pZH511	`aaaTTCgagaaaggtaccgcATG`	1,471	179.2	8.21

Table B. Bootstrap fitting GFP expression distributions measured by fluorescence microscopy. The convolution of the autofluorescence (pZH501) distribution and a log-normal distribution is fit to 100 bootstrapped samples of sizes identical to the experimental samples (samples of size N, sampled with replacement). This results in 100 estimates of the GFP expression mean (molecules/cell) and unitless noise (σ^2/μ^2). The mean and standard deviation of these estimates is reported here.

Strain	[ATc] (nM)	Mean (molecules/cell)	Noise	N
pZH509	0	283.3 +/- 4.2	0.0440 +/- 0.0059	484
	0.5	1763.3 +/- 28.0	0.0792 +/- 0.0099	751
	8	4124.3 +/- 44.4	0.0563 +/- 0.0048	733
	32	7989.8 +/- 114.8	0.0821 +/- 0.0069	719
pZH511	0	54.7 +/- 1.2	0.0868 +/- 0.0191	893
	0.5	234.7 +/- 5.3	0.0586 +/- 0.0098	369
	8	322.9 +/- 4.4	0.0795 +/- 0.0088	764
	32	671.1 +/- 8.3	0.0468 +/- 0.0051	560
pZH512	0	120.3 +/- 3.7	0.0705 +/- 0.0143	424
	0.5	600.6 +/- 8.2	0.0846 +/- 0.0077	702
	8	870.2 +/- 11.1	0.0712 +/- 0.0056	800
	32	1871.2 +/- 24.5	0.0696 +/- 0.0047	809

Supplemental Figures

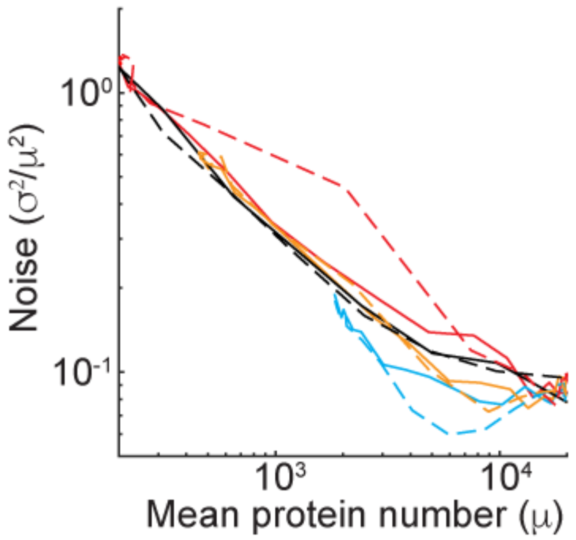

Fig A. Noise reduction for simulations in which free inducer numbers are held constant (solid lines; identical to **Fig 4C**) or where inducer is removed when bound to repressor (dashed lines) for constitutive expression (black), repressed expression (red), bicistronic autoregulated repression (cyan), and hybrid regulation (orange).

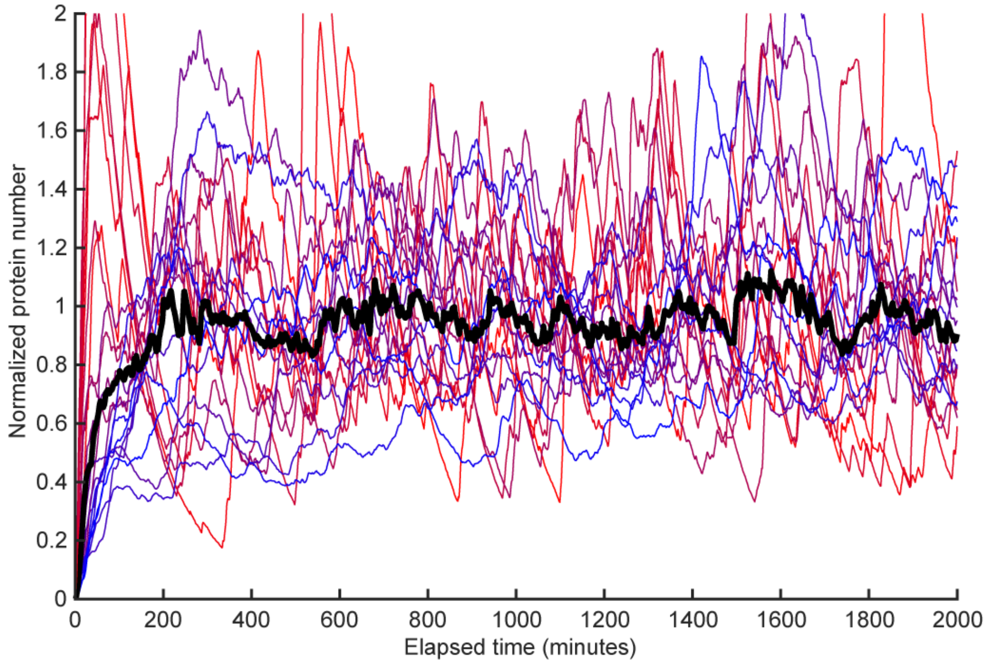

Fig B. First 2,000 minutes of simulated transgene expression for bicistronic, autoregulated construct at 21 different inducer levels ranging from 0 (red) to 524,288 molecules (blue), including extrinsic noise (data used for solid cyan lines in **Fig 1B** and **Fig 1C**). Protein levels are scaled by mean protein level calculated from time points from 1,000—101,000 minutes. The median of the 21 traces (thick black line) shows that equilibrium levels are typically reached after a few hundred minutes.

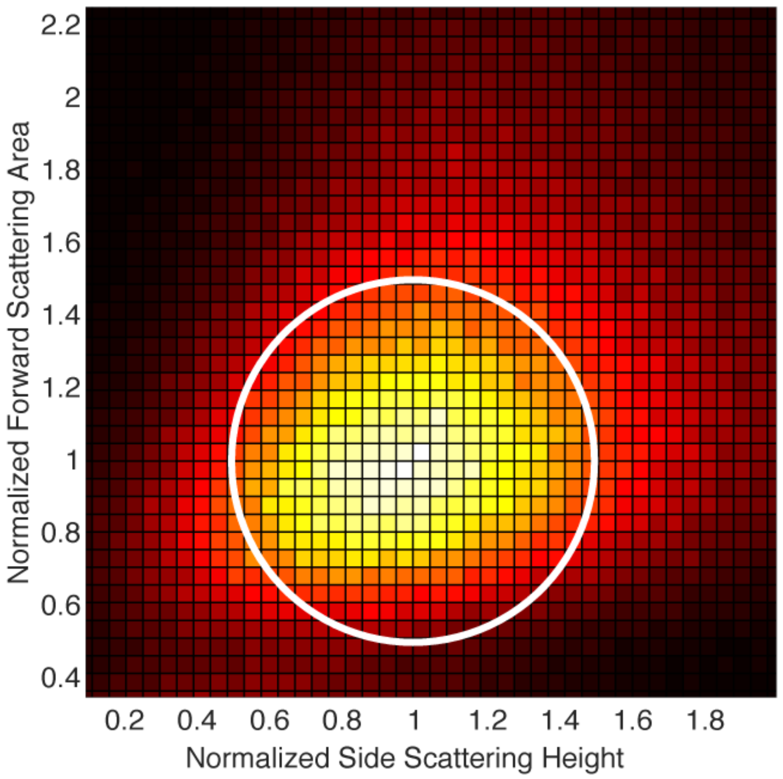

Fig C. Histogram of forward-scattering area and side-scattering height for all pZH509 data used for analysis underlying **Fig 2B**. The white circle indicates the gate used for selecting the >30% of cells in the neighborhood of the scattering peak.

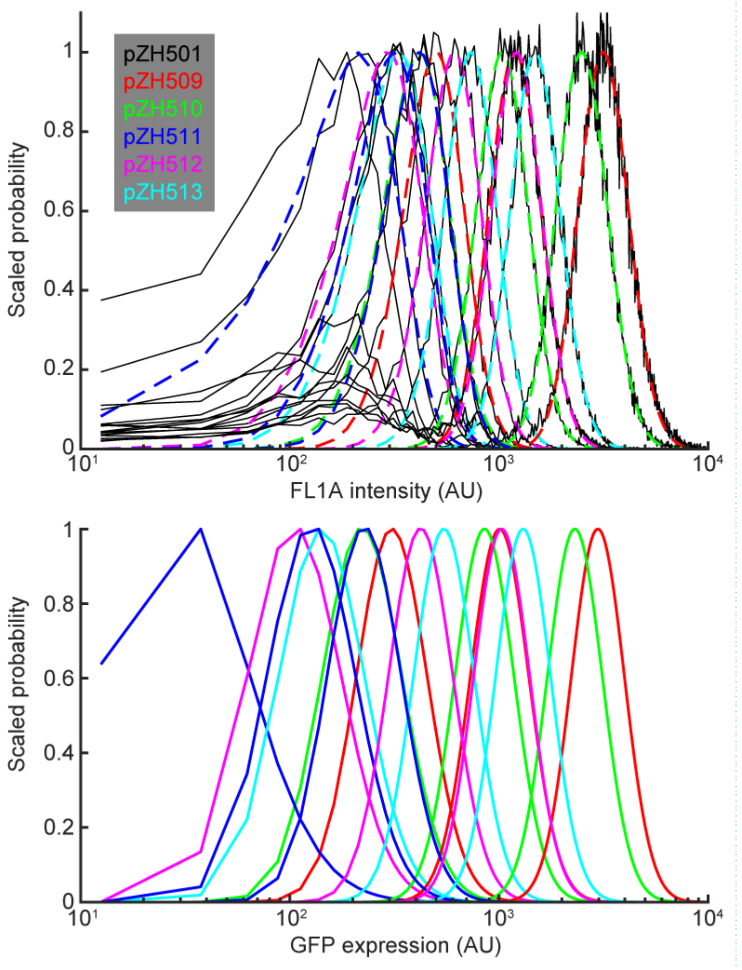

Fig D. Top, raw GFP flow cytometry histograms (black lines) used for analysis underlying **Fig 3B** with fits (dashed, lines colored as indicated for different plasmids) to convolution of the pZH501 distribution (no GFP, unfit black line) with log-normal distributions. Bottom, resulting log-normal distributions after fit; lines are colored according to strain identity with means monotonically increasing with ATc concentrations (2, 8, 32 nM).

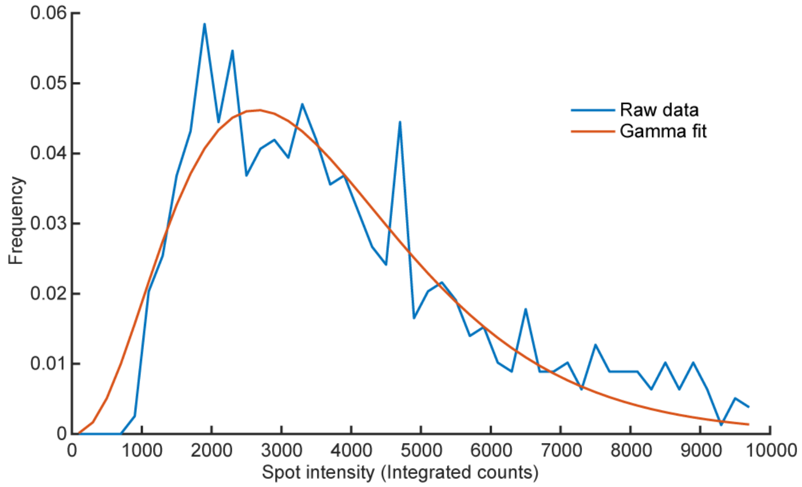

Fig E. Single-molecule spot intensity histogram (blue) fit to a gamma distribution (red) to estimate single-molecule intensities from GFP images at high laser power for pZH509 after photobleaching nearly all GFP molecules.

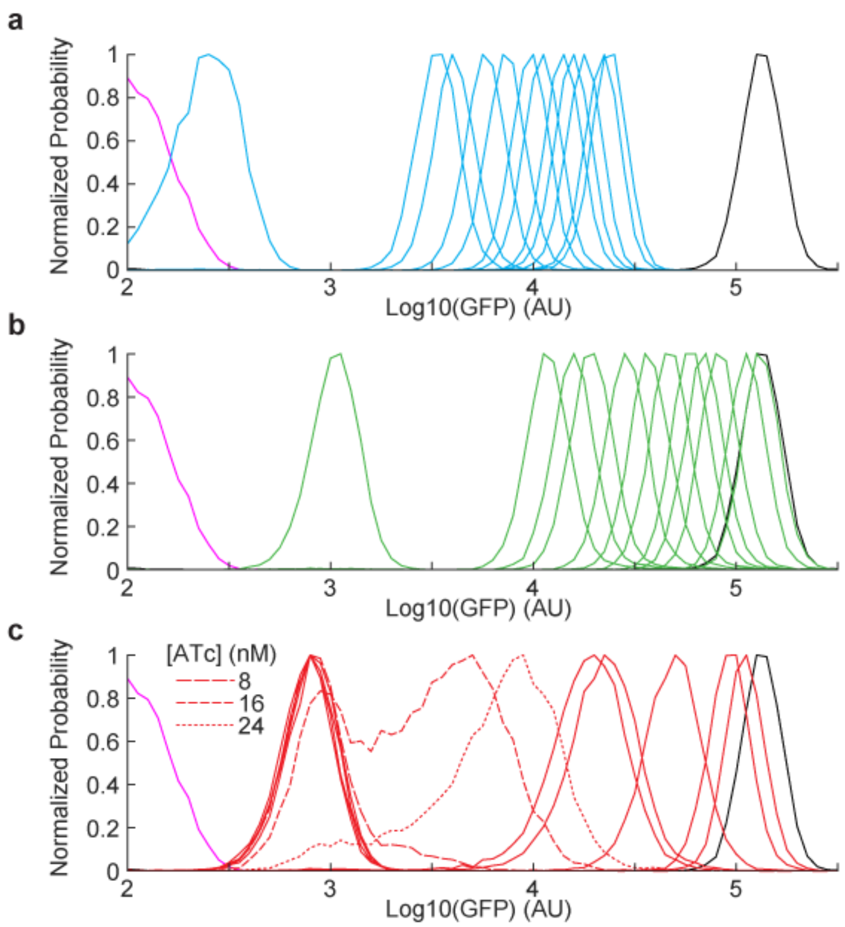

Fig F. Distribution of GFP fluorescence for alternative regulatory constructs measured by flow cytometry. Normalized probability of GFP fluorescence is shown for ATc concentrations of 0, 1, 2, 4, 8, 16, 24, 32, 40, 64, 128 and 256 nM (monotonic increase in expression with increasing ATc). (**a**) Bicistronic autoregulation plasmid pZH509. (**b**) Autoregulated repressor plasmid pZH517. (**c**) Constitutive repressor plasmid pZH520; three ATc concentrations are shown in differently dashed lines to show conditions giving high noise. For pZH520, [ATc] less than 8 nM gives very similar distributions.
